# Investigation of Behavior of Forced Degradation of Lidocaine HCl by NMR Spectroscopy and GC-FID Methods: Validation of GC-FID Method for Determination of Related Substance in Pharmaceutical Formulations 

**Published:** 2013

**Authors:** Yucel Kadioglu, Alptug Atila, Mehmet Serdar Gultekin, Nurdan Alcan Alp

**Affiliations:** a ***Department of Analytical Chemistry, Faculty of Pharmacy, Ataturk University, 25240, Erzurum, Turkey. ***; b ***Department of Chemistry, Faculty of Science, Ataturk University, 25240, Erzurum, Turkey. ***

**Keywords:** GC-FID, NMR, Lidocaine HCl, Forced degradation

## Abstract

The forced degradation study of lidocaine HCl was carried out according to the ICH guideline Q1A (R2). The degradation conditions were assessed to be hydrolysis, oxidation, photolysis and dry heat during 24 h, 48 h and 72 h and then the samples were investigated by GC-FID method and nuclear magnetic resonance (NMR) spectroscopy. According to these results, the degradation products were not observed in all reaction conditions during the 72 h period. Only spectral changes in the 1H and 13C-NMR spectrum were observed in hydrogen peroxide and acid degradation. As a result of this degradation, n-oxide was formed. After acid-induced degradation with HCl, the secondary amine salt was formed. Furthermore, trifluoroacetic acid (TFA) was used as the acidic media, and the decomposition products were observed. A simple and reliable gas chromatography method with flame ionization detection (GC-FID) was developed and validated for the determination of lidocaine HCl in pharmaceutical formulations in the form of a cream and injections. The GC-FID method can be used for a routine analysis of lidocaine HCl in pharmaceutical formulations and the proposed method, together with NMR spectroscopy, can be applied in stability studies.

## Introduction

Local anesthesia is described as any technique that induces the absence of sensation in part of the body, generally for the aim of inducing local analgesia, that is, local insensitivity to pain, although other local senses may be affected as well (1, 2). 

**Figure 1:**
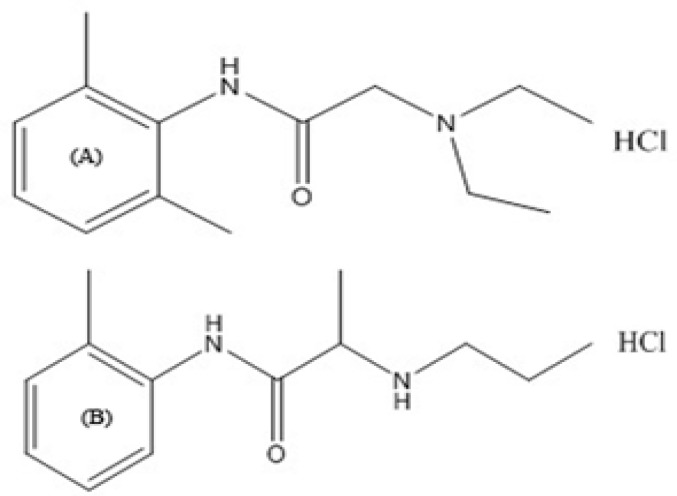
Chemical structure of lidocaine HCl (A) and IS [prilocaine HCl (B)].

In 1943, lidocaine was first synthesized under the name Xylocaine by Swedish chemist Nils Löfgren as an alternative class of anesthetics. Lidocaine HCl, 2-diethylamino-N-(2,6-dimethyl fenil)-etanamid hydrochloride (Figure 1 A), is a local anesthetic substance of the amide type (2). The analysis of lidocaine is important for keeping abreast of the patient’s medical process and quality controlling studies in pharmaceutical formulations. Because the anesthetic action of the local anesthetic substances is well known and appears to be especially useful on skin damaged by sun exposure, local anesthetic substances like lidocaine can be illegally present in cosmetic products, particularly in the formulation of after-sun cosmetics (2, 3). 

The literature shows that the thin-layer chromatography method (4), electroanalytical method (5), atomic absorption spectrophotometry method (6, 7), spectrophotometric methods (8- 13), HPLC methods with different detection (10, 13-19), capillary electrophoretic method (20) and membrane electrode method (21, 22) have been reported for determination of lidocaine HCl in pharmaceutical formulations. The determination of mixtures with other local anesthetics of lidocaine HCl in biological samples (23-28) and pharmaceutical formulation (29) have been done with the GC-MS and GC methods. There are only a few studies for determination of lidocaine HCl with the GC method in whole blood (30), serum (31), and urine (32). 

According to the current good manufacturing practices, the stability-indicating assay method before release must be assessed for all drugs. A forced degradation study of the drug substance can help to identify the possible degradation products, which can help to establish the degradation pathways and the intrinsic stability of the molecule and validate the stability-indicating power of the analytical procedures used (33). The nature of the forced degradation will depend on the individual drug substance and the type of the drug product involved (33). 

So far, according to our present knowledge, neither the GC-FID method for the analysis of lidocaine HCl alone in any pharmaceutical formulation nor the study of GC-FID and NMR spectroscopy for the degradation behavior of lidocaine HCl under various ICH-prescribed degradation conditions is available in the literature. There are a few studies about the stability of lidocaine HCl. These studies are described as follows: the relationship between pH and concentrations of antioxidants and vasoconstrictors in local anesthetic solutions (lidocaine and mepivacaine) (34), the short-term stability of lidocaine-adrenaline epidural solution in pH-adjusted solutions (35), the chemical stability of bupivacaine, lidocaine and epinephrine in pH-adjusted solutions (36), the stability of lidocaine and oxycodone in rectal gel with the HPLC method (37), the stability of lidocaine and epinephrine solutions exposed to electric current (38), the stability of lidocaine HCl and lidocaine HCl with epinephrine injectable solutions with the HPLC method (39), the stability with gradient HPLC-DAD of miconazole nitrate and lidocaine HCl in their combined oral gel dosage form (40) and finally in accordance with the study of George *et al.*, who exposed the four local anesthetics (lidocaine, mepivacaine, procaine and prilocaine) to standard autoclaving conditions (250 °C and 20 - 30 min) and analyzed the solutions with the gas chromatography method (41). 

The aim of the present work is to develop and validate a new GC-FID method for determination of lidocaine HCl in pharmaceutical formulations with a simple sample preparation using internal standard methodology and also to investigate the behavior of degradation of lidocaine HCl with the GC-FID method and NMR spectroscopy. The forced degradation studies were assessed according to the ICH guideline Q1A (R2) (33). The proposed method was validated with validation parameters, which are specificity, linearity, stability, analytical recovery, sensitivity, precision and accuracy in accordance with International Conference on Harmonization (ICH) guidelines (42). 

## Experimental

Lidocaine HCl that was used as reference substance and prilocaine HCl that was used as internal standard (IS; Figure 1 B) were purchased from Novagenix Bio Analytical R&D Centre (Ankara, Turkey). All other chemicals were purchased from Merck (Germany). All gases were supplied by Havas (Ankara, Turkey). 

The following pharmaceutical formulations of lidocaine HCl were obtained from local sources in Erzurum (Turkey): Emla^®^ Cream (5% cream, Astra Zeneca A.S., Turkey) containing 25 mg of lidocaine HCl, 25 mg of prilocaine HCl, 55 mg of poloxamer188 and 155 mg of poloxamer407. Jetokain^®^ Ampoule (2 mL ampoule, Adeka A.S., Turkey) containing about 20 mg of lidocaine HCl, 0.125 mg of epinephrine, 4.5 mg of sodium chloride and 1.0 mg of sodium metabisulfite per mL. Jetmonal^®^ Ampoule (2% 5 mL ampoule, Adeka A.S., Turkey) containing about 20 mg of lidocaine HCl and 4.6 mg of sodium chloride per mL. 

*Equipment*

The equipment used was: an Agilent 6890N Network gas chromatography system provided with an Agilent 7683 series auto sampler, a split/ splitless injector, flame ionization detector and Agilent chemstation software. 

^1^H and ^13^C NMR spectra were recorded on a Varian 400 spectrometer in deuterated chloroform (CDCl_3_), depending on the solubility of the product. Chemical shifts are reported in d ppm downfield with respect to tetramethylsilane as an internal standard in ^1^H and ^13^C NMR spectra. 

Photochemical experiments were performed in the glass tube of a photo reactor (RPR-200). UV radiation was obtained from 24 W, RPR (The Rayonet Photochemical Chamber Reactor) that irradiated at 350 - 400nm (λmax = 365 nm). The lamps were installed inside of the reactor and the entire system was placed in a closed box to avoid the disturbing effect of direct sunlight irradiation. 

*Chromatographic Conditions*

**Figure 2:**
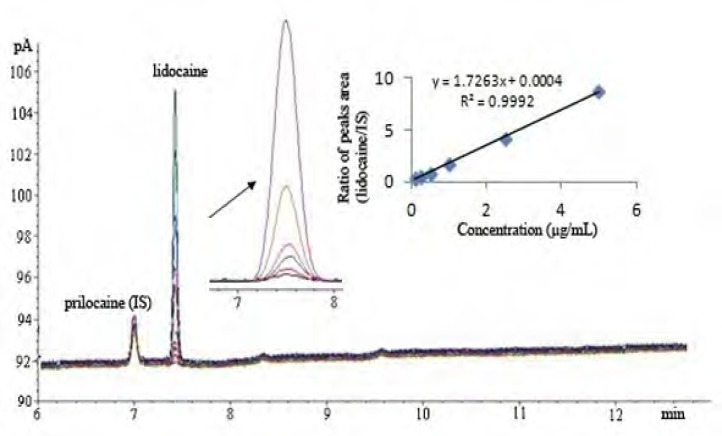
GC Chromatograms of standard solutions of lidocaine HCl and IS

Separation was achieved using a HP-5 capillary column (5% - phenyl -methylpolysilocone 30 m x 0.320 mm i.d., 25-μm film thickness, USA) with 2.0 μL of injector volume. The split mode (10:1) was used with nitrogen carrier gas and the flow rate of carrier gas was kept constant during analysis at 1.6 mL/min. Hydrogen and synthetic air were used as auxiliary gases for the detector (FID). The injector and detector temperatures were 260°C. The oven temperature programs were as follows: initial temperature of 80°C, held for 1.5 min at 210^o^C, ramp rate of 10°C/min and final temperature of 230°C, where the temperature was held for 0.5 min. The chromatograms under these circumstances were shown in Figure 2. 

*Preparations of standard solutions*

The standard working (SW) solutions (0.1, 0.25, 0.5, 1.0, 2.5 and 5.0 μg/mL) and quality control (QC) solutions (0.2, 1.25 and 4.0 μg/mL) were prepared in methanol from stock solution (100 μg/mL). 

SW solution of prilocaine HCl (IS) was prepared at 2.0 μg/mL concentration with methanol from the stock solution (50 μg/mL).

*Procedure of pharmaceutical formulations*

*Injections*

After the average volume for one ampoule was determined, the contents of ten ampoules for both injection samples were mixed into a volumetric flask. An aliquot of the solution equivalent to 20 mg lidocaine HCl was quantitatively transferred to a 50 mL-calibrated measuring flask and made up to the mark with methanol. The solutions were filtered through a 0.22-μm Millipore filter. The filtrate was diluted with methanol to obtain a 0.5 μg/mL concentration of lidocaine HCl for both injection samples.

*Cream*

An amount of the cream containing approximately 25 mg of lidocaine HCl was transferred with methanol to a centrifuge tube, and vortexed for 5 min. This solution was centrifuged for 5 min at 4000 rpm. The supernatant was filtered through a 0.22-μm Millipore filter into a volumetric flask. The filtrate was adjusted to volume with methanol to obtain a 0.5 μg/mL concentration of lidocaine HCl for the cream sample. 

About 2.0 μg/mL concentration of IS was added into the solutions prepared from cream and injections. The solutions were analyzed as described in section “Chromatographic Conditions”. 

## Results

*System suitability*

The system suitability tests were assessed by six replicate analyses of 5.0 μg/mL concentration of lidocaine HCl. In this study, the acceptance criterion was considered as ±2% deviation from the percent relative standard deviation (RSD %) of peak area and retention time of lidocaine HCl (Table 1). The number of theoretical plates (N) and the height of theoretical plate [(mm), mean (RSD %)] that was expressed as the efficiency of the column were found to be 186712.6 (2.56%) and 0.161 (1.56%), respectively, for injections of six replicate.

**Table 1:** System suitability study of method (5.0 μg/mL).

	**Retention time (min)** ^a^	**Peak area** ^a^
	7.53	22.58
SD	0.09	0.66
RSD %	1.23	2.92

^a^ based on six analysis, SD: standard deviation, RSD: relative standard deviation.

*Linearity*

Linearity of method was demonstrated over a linear range of 0.1- 5.0 μg/mL at six concentrations with 2.0 μg/mL concentration of IS (n = 6). The calibration curve was plotted by the ratio of the peak areas of lidocaine HCl and IS, versus the concentration of lidocaine HCl. The linear regression equation and statistical parameters was calculated by the least squares method using Microsoft Excel^®^ program and summarized in Table 2.

**Table 2:** Results of regression analysis of lidocaine HCl

**Parameters**	
Linearity (μg/mL)	0.1-50
Regression Equationa	by = 1.726x + 0.0004
Standard Deviation of Slope (Sa)	7.52 x 10^-7^
Standard Deviation of Intercept (Sb)	2.96 x10^-2^
Correlation Coefficient	0.9992
Standard Deviation of Correlation Coefficient	4.62x10^-3^
Relative Residual Standard Deviation (S_Δy/y, n-2_)	5.88x10^-2^
Limit of Detection (LOD, μg/mL)	0.03
Limit of Quantitation (LOQ, μg/mL)	0.09

^a^Average of six replicate determinations. ^b^ y: Peak-area ratios (lidocaine HCl/IS), x: lidocaine HCl concentration

*Sensitivity*

The limit of detection (LOD) is the lowest amount of analyte in a sample that can be detected but not necessarily quantitated as an exact value. The limit of quantitation (LOQ) is the lowest amount of analyte that can be quantitatively determined with suitable precision (42). The LOD and LOQ values were determined as 3:1 and 10:1 of the signal/noise ratio, respectively, and given in Table 2.

*Selectivity*

The selectivity of an analytical method may be defined as ability to unequivocally determine the analyte in the presence of additional components such as impurities, degradation products and matrix (42). The method of standard addition was used to demonstrate the effective separation of lidocaine HCl in the presence of excipients of pharmaceuticals and its degradation products. QC samples of lidocaine HCl were spiked into the solutions in equal amounts of six samples of 20 mg (for injections) and 25 mg (for cream) of lidocaine HCl and also IS was added into each sample. The samples were analyzed. Peak area ratios of lidocaine HCl and IS were measured for quantitative determination of lidocaine HCl. Any interference coming from the pharmaceutical formulations was observed. 

**Table 3:** Precision and accuracy of method

	**Within-day**			**Between-day**		
**Added** **( μg/mL )**	**Found**±**SDa** **( μg/mL )**	**Precision** ^b^ **RSD%**	**Accuracy** ^c^ **RE%**	**Found**±**SD**^a^ **( μg/mL )**	**Precision** ^b^ **RSD%**	**Accuracy** ^c^ **RE%**
0.20	0.19±0.002	1.05	-5.00	0.21±0.004	1.91	5.00
1.25	1.26±0.02	1.59	0.80	1.27±0.01	0.79	1.60
4.00	3.95±0.03	0.76	1.12	4.04±0.04	0.99	1.00

^a^ SD: standard deviation b RSD: relative standard deviation, ^c^ RE: relative error, (n = 6).

*Precision and accuracy*

Assay precision was determined by repeatability (within-day) and intermediate precision (between-day). Repeatability was evaluated by assaying six replicate analyses at the three concentrations during the same day. The intermediate precision was studied by analyzing the same samples on ten different days with the same procedure. The precision of the method was reported as the relative standard deviation [RSD % = (100 x standard deviation) / mean] and the accuracy of the method was given with percent relative error [RE % = (found concentration − known concentration) x 100 / known concentration]. The RSD % values for within-day and between-day precision for the proposed method were found to be ≤1.91% (n = 6). The RE % values for the within-day and between-day accuracy studies were found to be ≤5.0% (Table 3).

*Analytical recovery*

To double check the accuracy of the proposed method, the standard addition technique was applied. The QC solutions of standard sample were added to 0.5 μg/mL concentration of solutions of pharmaceutical formulations (injections and cream) and assayed with same procedure. The analytical recovery was calculated from the equation 1: 

Analytical Recovery % = [(C_t_-C_a_) / C_u_] x 100

(1)

Where Ct is total concentration of the analyte determined, Ca is the concentration of the pure analyte added to the formulation, and Cu is the concentration of the present analyte in the formulation. The average percent recovery values were determined approximately 97.07% and 99.03% for cream and injections samples, respectively, indicating good accuracy of the method. No interference from the common excipients was observed. The RSD % values of recovery studies were found as ranged from 0.89% to 4.60% (Table 4).

**Table 4:** Analytical recovery values by the standard addition method (n=6).

**Pharmaceutical Formulations**	**Taken Amount** **(μg/mL)**	**Added Amount** **(μg/mL)**	**Total Found Amount (μg/mL)** **(mean ±SD)**	**Recovery** **(%)**	**RSD** **(%)**
Jetokain® Ampoule	0.50	0.20	0.698±0.01	99.6	1.43
		1.25	1.741±0.08	98.2	4.60
		4.00	4.496±0.06	99.2	1.33
Jetmonal® Ampoule	0.50	0.20	0.696±0.02	99.2	2.87
		1.25	1.739±0.05	97.8	2.88
		4.00	4.501±0.08	100.2	1.78
Emla® Cream	0.50	0.20	0.684±0.01	96.8	1.46
		1.25	1.730±0.02	96.0	1.16
		4.00	4.492±0.04	98.4	0.89

*Hydrogen peroxide-induced degradation*

To study hydrogen peroxide-induced degradation, the drug solution (0.43 mmol) was prepared by dissolving 100 mg of powder lidocaine HCl in 50 mL methanol, and then 0.25 mL H_2_O_2_ (35%) was added to solution. This solution was kept at room temperature for 24 h, 48 h and 72 h in the dark brown glass containers and then the reaction mixture was evaporated to completely remove the excess of hydrogen peroxide under a nitrogen stream at room temperature. The residue was monitored by proton NMR spectroscopy. A new product was not observed in result of reaction. However, we observed only a small spectral change in 1H and 13C-NMR spectrum. We proposed that n-oxide was obtained in reaction conditions. 

^1^H-NMR (CDCl_3_, 400 MHz, ppm) *δ*=1.21 (t, J=6.9 Hz, 6H), 2.10 (s, 6H), 3.15 (q, J=6.9 Hz, 4H), 4.09 (brs, 2H), 7.26-6.91 (m, 3H).

^13^C-NMR (CDCl_3_, 100 MHz, ppm) *δ*=164.9, 135.4, 133.7, 128.2, 127.5, 52.5, 48.7, 18.6, 10.2.

*Acid-induced degradation with HCl*

The solution of lidocaine HCl (0.43 mmol) was heated to reflux temperature and 0.5 mL of concentrated (35%) hydrochloric acid solution was added slowly to this solution and stirred magnetically during 24 h, 48 h and 72 h at the same temperature. In addition, 1 M HCL was added into the lidocaine HCl solution and stirred magnetically during 24 h, 48 h and 72 h at reflux temperature. After the stirring process, the reaction mixture was neutralized by NaHCO_3 _solution and then extracted with ethyl acetate (3 x 25 mL). After that, the organic layer was evaporated and monitored by proton NMR spectroscopy. 

^1^H-NMR (CDCl_3_, 400 MHz, ppm) *δ*= 1.28 (t, J=7.2 Hz, 6H), 2.11 (s, 6H), 2.14 (q, J=7.2 Hz, 4H) 3.06 (brs, NH), 3.28 (m, 2H), 6.93-7.05 (m, 3H).

^13^C-NMR (CDCl_3_, 100 MHz, ppm) *δ*=163.7, 135.7, 135.4, 132.7, 128.2, 52.7, 49.7, 18.2, 9.4.

*Acid-induced degradation with trifluoroacetic acid (TFA)*

The solution of lidocaine HCl (0.43 mmol) was heated to reflux temperature and 1 mL of trifluoroacetic acid was added to this solution. The reaction mixture was stirred magnetically for 24 h and 48 h at the same temperature. After the stirring process, the mixture was evaporated, and the residue was purified with ethyl acetate: n-hexane mixture (3:7, v/v) on silica gel column chromatography. The final products were determined by NMR spectroscopy.

*2,5-dimethyl aniline*

^1^H-NMR (CDCl_3_, 400 MHz, ppm) *δ*=2.10 (s, 6H), 7.25-6.92 (m, 3H),

^13^C-NMR (CDCl_3_, 100 MHz, ppm) *δ*= 135.2, 133.2, 128.3, 127.8, 18.3.

*2-(diethylamino)acetic acid*

^1^H-NMR (CDCl_3_, 400 MHz, ppm) *δ*=1.25 (t, J=6.8 Hz, 6H), 3.23 (brq, J= 6.8 Hz, 4H), 4.17 (brs, 2H) 10.01 (brs, 1H). ^13^C-NMR (CDCl3, 100 MHz, ppm) *δ*=163.5, 52.0, 48.9, 9.3

*Base-induced degradation studies*

In the study in alkaline condition, the solution of lidocaine HCl (0.43 mmol) was heated to reflux temperature and 2.5 mL of 1.0 M NaOH solution was added slowly to this solution. The reaction mixture was stirred magnetically for 24 h, 48 h and 72 h at the same temperature. After the stirring process, the mixture was neutralized by 1.0 M HCl solution and then diluted. The reaction mixture was extracted with ethyl acetate (3 x 25 mL), and the organic layer was evaporated. The residue was monitored by proton NMR spectroscopy. The degradation products were not observed. 

*Dry heat degradation*

In order to observe the effect of temperature on degradation of lidocaine HCl, 50 mg (0.215 mmol) powder lidocaine HCl was exposed to dry heat at 120°C and kept for 24 h, 48 h and 72 h at same temperature. After that, the residue was cooled to room temperature and monitored by proton NMR spectroscopy. The degradation products were not observed.

*Photolytic degradation*

In the photolytic stability study, two solutions were prepared in acetone. The first solution includes 100 mg (0.215 mmol) of lidocaine HCl and the second solution was prepared by adding 5 mg of *p*-hydroquinone, used as a sensitizer solution, and 100 mg of lidocaine HCl into 25 mL of acetone. To start the experiments, the lamps of the photo reactor (RPR) were turned on and warmed up for about 10 min. After that, the prepared solutions were placed into the reactor’s glass tube and then the photochemical reaction at room temperature was started and exposed to light (365 nm) during 24 h, 48 h and 72 h. Under illumination, the reaction temperature was kept at 20 - 25°C. After this process, the reaction solution was evaporated and the residue was purified on the silica gel column chromatograph. The reaction mixture was monitored by proton NMR spectroscopy. Any degradation products were assessed.

*Application of the method for analysis of pharmaceutical formulations*

The proposed method was evaluated in the assay of commercially available a cream containing 25 mg of lidocaine HCl and two brands of injections containing 20 mg of lidocaine HCl. Assessment was created using the calibration curve method. Any important difference between the slopes of the calibration curves of pharmaceutical formulation and standard solutions was observed. The accurately weighted amounts of ampoules and cream including 20 mg and 25 mg of lidocaine HCl, respectively, were determined (n = 6). 

The results obtained are satisfactorily accurate and precise, as indicated by the excellent % recovery and SD <1.21 (Table 5). Experiments showed that there was no interference from the additions and excipients. In the determination repeated six times, mean recovery for all formulations obtained approximately 99.93%, with an RSD% <5.54%. 

**Table 5:** Determination of lidocaine HCl in pharmaceutical formulations

**Pharmaceutical Formulations**	**Label Claim**	^a^ **Mean±SD**	**Recovery (%)**	**RSD (%)**
Jetokain ^®^ ampoule	20 mg per mL	19.98±1.21	99.9	6.06
Jetmonal ^® ^ampoule	20 mg per mL	19.96±1.12	99.8	5.61
Emla^®^ cream	25 mg	20.01±0.99	100.1	4.95

^a^Average of six replicate determinations

## Discussion

**Figure 3:**
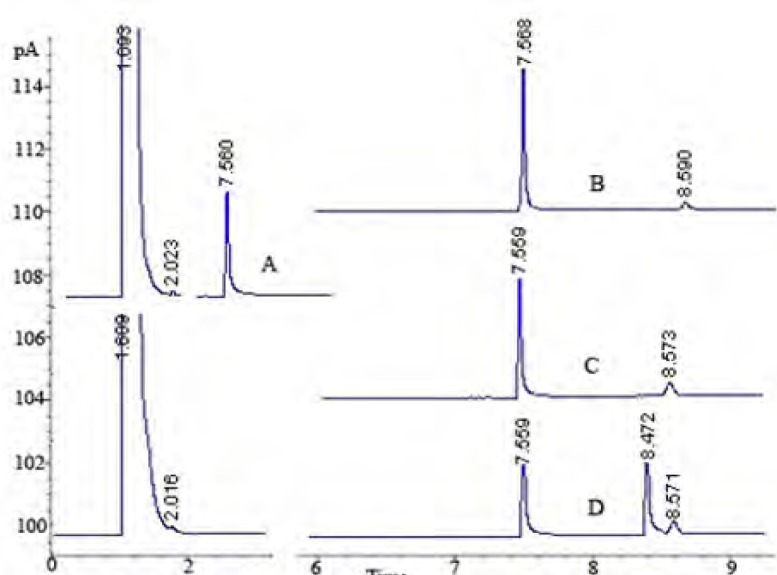
GC Chromatograms: A: the standard solution of lidocaine HCl; B: degradation with HCl for 72 h; C: degradation with H_2_O_2_ for 72 h; D: degradation with trifluoroacetic acid (TFA) for 72 h

**Figure 4:**
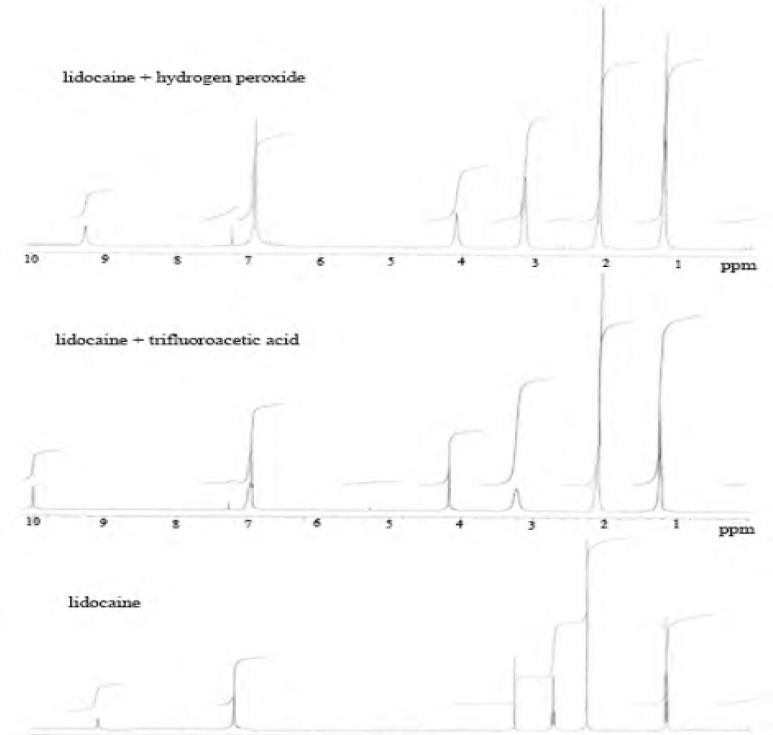
Proton NMR spectrums of lidocaine HCl and degradation with H_2_O_2_ and trifluoroacetic acid (TFA).

In the pharmaceutical industry, the disciplines primarily involved with stability are pharmaceutical analysis and product development. The requirement of the establishment of a stability-indicating assay has become more clearly mandated with the International Conference on Harmonization (ICH) guidelines (33). The guidelines explicitly require conduct of forced degradation studies under a variety of conditions, like pH (acid-base), photolytic, oxidation and dry heat (33, 43). Understanding drug degradation in formulated product is important in pharmaceutical development as drug stability and degradation products could have significant impacts on formulation development, analytical method development, package development, storage conditions, shelf-life determination, and safety and toxicology concerns (43). Consequently, we aimed to designate the GC-FID method for indication of the stability-indicating properties of lidocaine HCl, and the forced degradation studies were performed under various stress conditions. The suitability of the proposed method as a stability- indicating method was supported by NMR studies. After the forced degradation process, final products were diluted with methanol to obtain 4 μg/mL solutions and injected into the GC system. The chromatograms obtained are presented in Figure 3. In addition, the degradation reaction mixture was monitored by NMR spectroscopy (Figure 4). 

According to the degradation results, it was observed that lidocaine HCl has a very stable skeleton structure. In acidic conditions, when lidocaine HCl was treated with HCl acid, it was observed that secondary amine salt of lidocaine was formed to yield 100%. In proton NMR spectroscopy, resonance structure was produced as multiple of doublet in 3.28 ppm. Since, each of CH_2_ protons has the site of different spaces. 

**Scheme 1:**
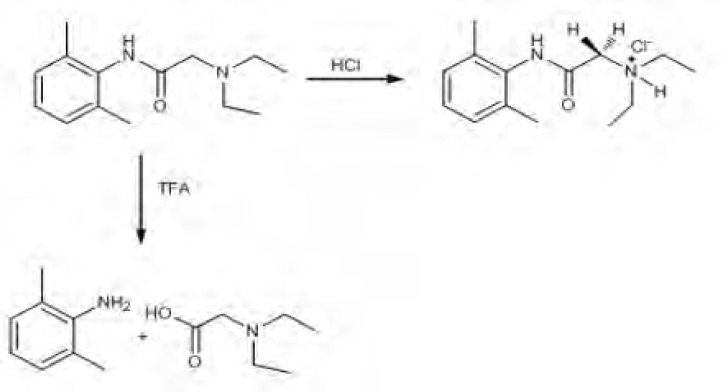


It was observed that proton NMR signals obtained were the same with molecules of similar nature in the literature (44). When trifluoroacetic acid (TFA) was used as the acidic media, as different from International Conference on Harmonization (ICH) guidelines, decomposition products were observed (Scheme 1). 

Oxidation of lidocaine HCl with H_2_O_2_ was done at room temperature, and a small change in ^1^H-NMR spectrum of lidocaine was observed as a result of the oxidation reaction. Hence, we proposed that nitrogen groups in the structure of lidocaine HCl were oxidized with H_2_O_2_ as n-oxide. In alkali conditions, skeleton structure of lidocaine HCl was protected. After dry heat and photolytic studies we determined that lidocaine HCl was very stable against heat conditions (120 ^o^C) and light (365 nm) over 72 h. 

## Conclusions

A rapid and sensitive gas chromatography method with flame ionization detection (GC-FID) was developed and validated for the determination of lidocaine HCl in pharmaceutical formulations in the form of cream and injections. The proposed method has several advantages, which are high specificity, good accuracy and precision values, short chromatographic run time (8.5 min) and high analytical recovery value. In forced degradation studies, many forms of degradation and fragmentation were not observed in destruction reactions. It was found that the structure of the lidocaine HCl molecule was very stable in alkaline, acidic, oxidation, dry heat and photolytic media. Furthermore, trifluoroacetic acid (TFA) was used as the acidic media. In this media, the decomposition products occurred. The developed GC-FID method, together with NMR spectroscopy, for determination of lidocaine HCl in pharmaceutical formulations can be used for both routine analysis in quality control studies and analysis of degradation products during the stability studies. 
